# Endovascular thrombectomy for large-core stroke: a meta-analysis with trial sequential analysis

**DOI:** 10.1055/s-0045-1806827

**Published:** 2025-05-13

**Authors:** Marco Antonnio Rocha dos Santos, Pierludovico Moro, Abner Lucas Balduino de Souza, Lauren Nirta, Thaís Pereira Mendes, Laura de Lima Xavier, Ming-Chieh Ding

**Affiliations:** 1 Universidade do Planalto Catarinense, Lages SC, Brazil.; 2 Università La Sapienza, Department of Human Human Neuroscience, Roma LAZ, Italy.; 3 Universidade Evangélica de Goiás, Anápolis GO, Brazil.; 4 MediSpeak Communications, Busan, South Korea; 5 Department of Psychiatry and Legal Medicine, Institute of Psychiatry, Universidade Federal do Rio de Janeiro, Rio de Janeiro, RJ, Brazil; 6 Washington University in St. Louis, St. Louis MO, United States.; 7 University of North Carolina, School of Medicine, Division of Stroke and Vascular Neurology, Chapel Hill NC, United States.

**Keywords:** Diagnostic Imaging, Ischemic Stroke, Thrombectomy, Mechanical Thrombolysis, Patient Outcome Assessment

## Abstract

**Background:**

Recent studies have reported that endovascular thrombectomy (ET) may improve neurological outcomes in large-core stroke.

**Objective:**

We performed a systematic review and meta-analysis to compare the pooled efficacy and safety of ET and of the best medical treatment among patients with large-core stroke.

**Methods:**

We searched the PubMed/MEDLINE, Scopus, and Cochrane databases from inception to November 2023. The inclusion criteria were randomized controlled trials (RCTs) comparing ET and the best medical treatment available among patients with large-core stroke (Alberta Stroke Program Early Computed Tomography Score [ASPECTS] < 6 or ischemic core > 50 mL on perfusion imaging) within 24 hours of symptom onset.

**Results:**

We included 6 RTCs comprising 1,887 patients (ET group: *n* = 945). Endovascular thrombectomy was associated with good neurological outcomes (odds ratio [OR]: 2.92; 95% confidence interval [95%CI]: 2.17–3.93), or independent walking (OR: 2.22; 95%CI: 1.72–2.86). Trial sequential analysis confirmed a robust statistical significance for good neurological outcomes favoring ET. Endovascular thrombectomy was associated with higher risks of developing intracranial bleeding (OR: 2.65; 95%CI: 1.35–5.22) and symptomatic intracranial bleeding (OR: 1.83; 95%CI: 1.14–2.94). There were no differences between the groups regarding mortality or decompressive craniectomy. Patients submitted to non-contrast computed tomography (CT) with CT angiography (CTA) scans were analyzed separately and showed good neurological outcomes, comparable to those of the patients submitted to other imaging modalities (OR: 3.24; 95%CI: 1.52–6.92).

**Conclusion:**

Endovascular thrombectomy was associated with good neurological outcomes and independent walking in patients with large-core acute ischemic stroke. However, it was also associated with an increased risk of developing intracranial bleeding. Non-contrast head CT with CTA scans may be appropriate for screening patients to undergo ET.

## INTRODUCTION

Endovascular thrombectomy (ET) is the established treatment for ischemic stroke caused by large vessel occlusion (LVO); however, its effectiveness in the treatment of large-core strokes remains unclear. 1 2

Several randomized controlled trials (RCTs) 3 4 5 6 and systematic reviews 7 8 have demonstrated the superiority of ET compared with the best medical treatment (BMT) for ischemic stroke due to LVO. Recent RCTs 9 10 11 12 13 14 have investigated the viability of ET for large-core stroke (defined as an Alberta Stroke Program Early Computed Tomography Score [ASPECTS] < 6 or ischemic core > 50 mL on perfusion imaging) and reported promising results. Two systematic reviews 15 16 were recently performed using data from these RCTs. One study 15 limited their analysis to a treatment window of 6 hours from symptom onset, and the other 16 examined only functional independence (score on the Modified Rankin Scale [mRS] ≤ 2 at 90 days), mortality, and intracerebral hemorrhage, and did not perform subgroup analyses. 16 Few reviews have included the results from all the 6 RCTs as well as a relevant subgroup analysis.

Due to the recent publication of new important RCTs, 9 10 11 12 13 14 we conducted a meta-analysis to conclusively determine the efficacy and safety of ET for the treatment large-core stroke. Subgroup analyses were performed based on neuroimaging modality, ASPECTS classification, and treatment window (≤ 6 hours and > 6 hours from symptom onset), and supplemented by trial sequential analysis (TSA) to gauge the benefits of ET in the treatment of large-core stroke.

## METHODS

The current systematic review and meta-analysis investigated the pooled efficacy and safety of ET versus BMT among patients with large-core stroke by analyzing the following outcomes of interest: good neurological outcome, defined as an mRS score ≤ 2; independent walking, defined as an mRS score ≤ 3; early neurological improvement (ENI), defined according to the specifications of the studies; 9 10 11 12 13 14 90-day mortality; decompressive craniectomy; intracranial bleeding; and symptomatic intracranial bleeding.

The present study was registered *a priori* in the International Prospective Register of Systematic Reviews (PROSPERO; registration number: CRD42023493212), and reporting was guided by the Preferred Reporting Items for Systematic Reviews and Meta-Analyses (PRISMA) statement. 17

### Search strategy

We conducted a literature search on the PubMed/MEDLINE, Cochrane, and Scopus databases on November 28, 2023. The search strategy followed a systematic review methodology and included synonyms and related terms based on the patient population of interest ( *large infarct* OR *large ischemic stroke* OR *large ischemic region* OR *large ischemic core* OR *large anterior* *circulation ischemic stroke* ) and the intervention of interest ( *endovascular thrombectomy* OR *mechanical thrombectomy* OR *mechanical revascularization* ). The Boolean operators *AND* and *OR* were used to combine search terms and refine the search results. The search included all studies published until the search date, with no language restrictions. The search strategy was adapted for each database as necessary.

### Literature search and eligibility

Two investigators (MARS and PM) independently appraised the titles and abstracts of the initial search results and selected studies for full-text review and screening. The inclusion criteria were: studies conducted in adults (aged >18 years) diagnosed with large-core stroke (ASPECTS < 6 or ischemic core > 50 mL on perfusion imaging) who presented within the first 24 hours of symptom onset; studies investigating ET as the intervention and BMT as the control; and RCTs reporting at least one outcome of interest. And the exclusion criteria were: non-original articles; gray literature; duplicate publications; studies with no imaging criteria specified; and studies that were stopped early based on the results of other studies.

### Data extraction

Data was extracted and validated manually by two independent reviewers (MARS and PM) using a data extraction tool developed specifically for the current study ( **Supplementary Material 1** –available at https://www.arquivosdeneuropsiquiatria.org/wp-content/uploads/2025/02/ANP-2024.0193-Supplementary-Material-1.xlsx ; online only). Conflicts were resolved by consensus.

### Quality assessment

Two authors (MARS and PM) independently performed the risk of bias evaluation using the Cochrane risk-of-bias tool for randomized trials (RoB2), 18 with conflicts resolved by consensus. Publication bias was assessed using funnel plot analyses for good neurological outcome and intracranial bleeding endpoints.

### Data analysis

The treatment effects for binary endpoints were compared using pooled odds ratios (ORs) and 95% confidence intervals (95%CIs). The random effects model was used for all outcomes regardless of heterogeneity, considering that methodological and sample differences were anticipated. Heterogeneity was examined through the Cochran's Q test, I ^2^ statistics, and Tau-square using the restricted maximum-likelihood method. 19 We performed an ordinal shift analysis including data from each included study using the Wilcoxon test to analyze the differences between groups across the entire mRS score range (0 indicating asymptomatic and 6 indicating death) at 90 days. 20 All statistical analyses were performed using the R statistical software (R Foundation for Statistical Computing, Vienna, Austria), version 4.3.2.

The TSA was performed for the good neurological outcome endpoint using the Trial Sequential Analysis software (Copenhagen Trial Unit, Copenhagen, Denmark), version 0.9.5.10 Beta. Diversity-adjustment was calculated using a 2-sided alpha of 0.05, a beta of 0.01 (power of 99%), and an event proportion of 20% in the ET group and of 8% in the BMT group. The cumulative Z curve was constructed using a random effects model. The TSA findings in which the 95%CI boundaries did not include the null (< 1.00 or > 1.00) were considered statistically significant. The anticipated relative risk and the event proportion in the BMT group were calculated 21 22 based on the results of the current meta-analysis.

### Sensitivity analysis

Sensitivity analyses were performed for outcomes with moderate or high heterogeneity using the leave-one-out approach. L'Abbé 23 and Baujat et al. 24 plots were developed and evaluated to identify studies which substantially contributed to heterogeneity.

## RESULTS

### Study selection and baseline characteristics

**Figure 1:**
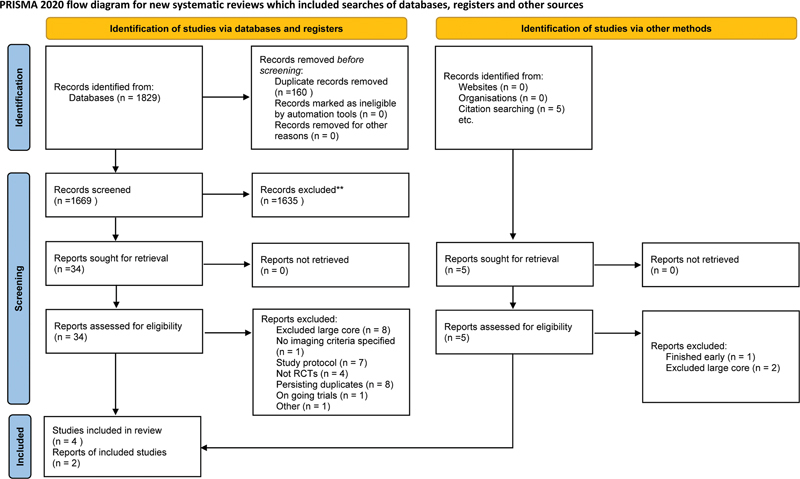
Preferred Reporting Items for Systematic Reviews and Meta-Analyses 17 (PRISMA) flow diagram.

The initial database search yielded 1,829 potential articles. One study 25 was excluded despite containing some patients that could be included in the current systematic review, as it was not powered for outcome comparisons and was not equally randomized for low ASPECTS. Ultimately, 6 RCTs 9 10 11 12 13 14 were included in the present systematic review and meta-analysis ( Figure 1 ), with a total of 1,887 patients (ET group: *n* = 945; and BMT group: *n* = 942). The characteristics of the studies are presented in Table 1 .

**Table 1:** Baseline characteristics of the included studies

Study	TENSION, 2023 13	RESCUE-Japan LIMIT 2022 9	LASTE, 2024 14	SELECT2, 2023 10	ANGEL-ASPECT, 2023 11	TESLA, 2023 12
**Patients:** **ET/BMT**	125/128	101/102	159/165	178/174	230/225	152/148
**Male – n (%):** **ET/BMT**	59 (55)/51 (48)	55(54.5)/58 (56.9)	170 (52.47)/154 (47.53)	107 (60.1)/100 (57.5)	135 (58.7)/144 (64)	76 (50)/84 (56.8)
**Age (in years):** ET/BMT ^*^	73 (65–81)/74 (64–80)	76.6 (66.6–86.6)/ 75.7(65.5–85.9)	73 (66–79)/74(65–80)	66 (58–75)/67 (58–75)	68 (61–73)/67 (59–73)	66 (54–74)/67.5 (57.5–73.5)
**Race or ethnic group – n (%):** **ET/BMT**	NA	Asian: 101 (100)/102 (100)	NA	Native American: 0/1 (0.6); Asian: 5 (2.8)/3 (1.7); White: 132 (74.2)/130 (74.7); Native Hawaiian: 2 (1.1)/0; other: 13 (7.3)/16 (9.2)	Asian: 230 (100)/225 (100)	Asian: 1 (0.7)/5 (3.4); Black: 32 (21.1)/34 (23); White: 99 (65.1)/92 (62.2); Hispanic or Latino: 12 (7.9)/16 (10.8) Native Hawaiian: 1 (0.7)/0; other, unknown, or declined to answer: 7 (4.6)/1 (0.7)
** ASPECTS: ^§^** ET/BMT ^*^	4 (3–5)/4 (3–5)	3 (3–4)/4 (3–4)	2(1–3)/2(1–3)	4 (3–5)/4 (4–5)	3 (3–4)/3 (3–4)	4 (3–5)/4 (3–5)
**Baseline** **NIHSS score:** ET/BMT ^*^	19 (16–22)/8 (15–22)	22 (18–26)/22 (17–26)	21(18–24)/21(18–24)	19 (15–23)/19 (15–22)	16 (13–20)/15 (12–19)	19 (15–23)/18 (14,5–21)
**IV thrombolysis – n (%):** **ET/BMT**	49 (39/44 (34)	27 (26.7)/29 (28.4)	55 (34.6)/58 (35.8)	37 (20.8)/30 (17.3)	66 (28.7)/63 (28.0)	31 (20.4)/30 (20.3)
Time from stroke onset to randomization (in hours): ET/BMT ^*^	2 (1.2–3.5)/2.1(1.2–3.6)	3.82 (2.4–7.65)/3.57 (2.37–6.3)	4.51(3.31–5.85)/4.46(3.45–5.6)	9.17 (5.31–15.42)/9.60 (5.78–15.20)	7.55 (4.98–11.86)/7.71 (5.08–13.01)	10.42 (5.55–15.69)/12.39 (5.46–17.1)
**< 6 hours from onset –n (%):** **ET/BMT**	78 (62.4)/82 (64.1)	71 (70.3)/77 (72.6)	NA	NA	82 (35.7)/85 (37.8)	42 (27.6)/41 (27.7)
**6 to 12 hours from onset – n (%):** **ET/BMT**	47 (37.6)/46 (35.9) ^**^	18 (17.8)/13 (12.7)	NA	NA	92 (40.0)/76 (33.8)	NA
**> 12 hours from onset – n (%):** **ET/BMT**	0/0	12 (11.9)/12 (14.7)	Zero	NA	56 (24.3)/64 (28.4)	NA
**M2 occlusion – n (%):** **ET/BMT**	0/125 (0)/1/127 (1)	0/3 (2.9)	NA	7 (3.9)/8 (4.6)	2 (0.9)/2 (0.9)	3 (2.1)/10 (6.9)
**M1 occlusion – n (%):** **ET/BMT**	83 of 125 (66)/88 of 127 (69)	74 (73.3)/70 (68.6)	88 (55.3)/91 (55.2)	91 (51.1)/100 (57.5)	145 (63.0)/142 (63.1)	97 (66.4)/94 (64.8)
**ICA occlusion – n (%):** **ET/BMT**	41 of 125 (33) / 37 of 127 (29) ^‡^	47 (46.5) /49 (48.0)	69 (43.4)/74 (44.8)	80 (44.9)/66 (37.9)	83 (36.1)/81 (36.0)	46 (31.5)/41 (28.3)
** Aspiration catheter alone: ^†^** ** n (%) ^¶^**	21 of 121 (17) ^‡^	11 of 98 (11.2) ^‡^	NA	NA	56 of 230 (24.3)	NA
** Stent retriever alone: ^†^** ** n (%) ^¶^**	28 of 121 (23) ^‡^	9 of 98 (9.2) ^‡^	NA	NA	41 of 230 (17.8)	NA
**Aspiration catheter and** ** stent retriever: ^†^** ** n (%) ^¶^**	72 of 121 (60) ^‡^	78 of 98 (79.6) ^‡^	NA	NA	115 of 230 (50.0)	NA
**Imaging used toselect patients** ** (%) ^¶^**	CT/CTA (82.2%) or MRI/MRA (17.8%)	MRI/MRA (86.2%) or CT/CTA (14.8%)	MRI/MRA (83.6%) or CT/CTA (16.4%)	CT plus CTP (98%), CT plus MRI (2%);ischemic core >50mLon perfusion imaging)	CT/CTA or MRA. If ASPECTS < 2 or > 5, CTP was performed (70–100mL of core)	CT/CTA only

Abbreviations: ASPECTS, Alberta Stroke Program Early Computed Tomography score; BMT, best medical treatment; CT, computed tomography; CTA, computed tomography angiography; CTP, computed tomography perfusion; ET, endovascular thrombectomy; ICA, internal carotid artery; IV, intravenous; M1, first main branch of the middle cerebral artery; M2, second main branch of the middle cerebral artery; MRA, magnetic resonance angiography; MRI, magnetic resonance imaging; NA, not available; NIHSS, National Institutes of Health Stroke Scale.

Notes: *Median (interquartile range, IQR). ^§^ Value based on CT or MRI scans. **Six to 11 hours. ^†^ ET technique. ^¶^ When available. ^‡^ Only for available data.

### Efficacy outcomes

**Figure 2:**
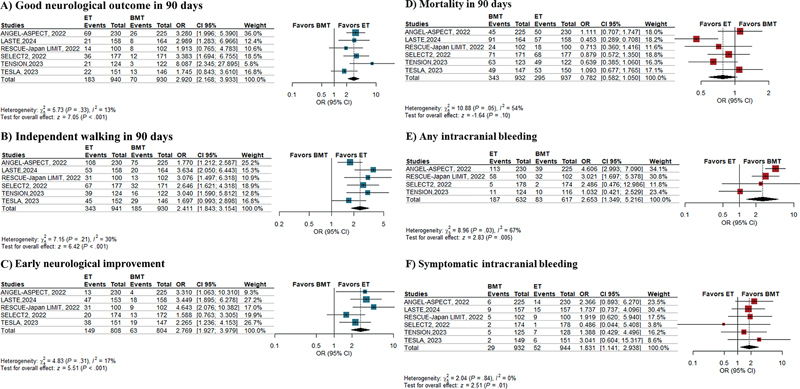
Abbreviations: 95% CI, 95% confidence interval; BMT, best medical treatment; ET, endovascular thrombectomy; OR, odds ratio. Forest plots of efficacy and safety outcomes.

Endovascular thrombectomy was associated with good neurological outcomes (mRS score ≤ 2; OR: 2.92; 95%CI: 2.17–3.93; Figure 2A ), independent walking (mRS score ≤ 3; OR: 2.41; 95%CI: 1.84–3.15; Figure 2B ), and ENI (OR: 2.76; 95%CI: 1.93–3.98; Figure 2C ).

**Figure 3:**
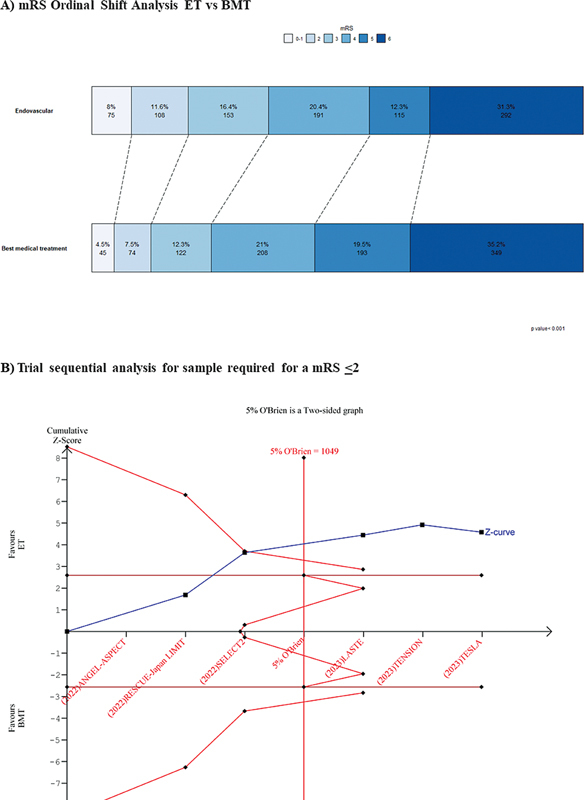
( **A** ) Ordinal shift analysis of the scores on the Modified Rankin Scale (mRS; endovascular thrombectomy [ET] versus best medical treatment [BMT]). ( **B** ) Trial sequential analysis of the sample required for good neurological outcomes (mRS score ≤ 2).

The ordinal shift analysis of the mRS scores was performed with data from the 6 RCTs 9 10 11 12 13 14 included, and it showed a significant difference between the BMT (median = 5; interquartile range [IQR]: 3–6) and ET (median = 4; IQR: 1–6) groups ( *p* < 0.001). Endovascular thrombectomy was also associated with a higher proportion of patients with good neurological outcomes (ET group: 183/934 [19.6%] versus BMT group: 119/991 [12%]) and independent walking (ET group: 336/934 [35.97%] versus ET group: 241/991 [24.31%]) ( Figure 3A ).

For the TSA, the cumulative Z curve crossed the trial sequential monitoring boundary for benefit ( Figure 3B ), and the total sample size surpassed the estimated information size of 5% of the 1,049 patients. This outcome indicates that the sample size was higher than needed, suggesting a probable true positive.

### Safety outcomes

There was no difference between the BMT and ET groups in terms of 90-day mortality (OR: 0.78; 95%CI: 0.582–1.050; Figure 2D ) or the incidence of decompressive craniectomy (OR: 1.19; 95%CI: 0.78–1.83). The ET group experienced more intracranial bleeding (OR: 2.65; 95%CI: 1.35–5.22; Figure 2E ) and symptomatic intracranial bleeding (OR: 1.83; 95%CI: 1.14–2.94; Figure 2F ). Due to the high heterogeneity in the intracranial bleeding outcome (I ^2^ = 67%), we performed a leave-one-out sensitivity analysis ( **Supplementary Material 2** , Figure S1 – available at https://www.arquivosdeneuropsiquiatria.org/wp-content/uploads/2025/02/ANP-2024.0193-Supplementary-Material-2.docx ; online only). The removal of the Study of Endovascular Therapy in Acute Anterior Circulation Large Vessel Occlusive Patients with a Large Infarct Core 11 (ANGEL-ASPECT; OR: 2.01; 95%CI: 0.93–4.34; I ^2^ = 49%) and the Recovery by Endovascular Salvage for Cerebral Ultra-Acute Embolism Japan Large Ischemic Core Trial 9 (RESCUE-Japan LIMIT; OR: 2.4; 95%CI 0.88–6.59; I ^2^ = 77%) reduced the frequency of intracranial bleeding in the ET group compared with the bmt group, but did not eliminate the heterogeneity in the endpoint. The Baujat et al. 24 plot confirmed that the heterogeneity in this endpoint was predominantly from the ANGEL-ASPECT and the Efficacy and Safety of Thrombectomy in Stroke with Extended Lesion and Extended Time Window 13 (TENSION; omitting TENSION did not influence the outcome). The L'Abbé 23 plot confirmed that the ANGEL-ASPECT and RESCUE-Japan LIMIT were responsible for the higher event rate ( **Supplementary Material 2** , **Figures S2** – **S3** ; online only). Neurological deterioration was reported by three trials (A Randomized Controlled Trial to Optimize Patient's Selection for Endovascular Treatment in Acute Ischemic Stroke [SELECT2], 10 Thrombectomy for Emergent Salvage of Large Anterior Circulation Ischemic Stroke 12 [TESLA], and Large Stroke Therapy Evaluation 14 [LASTE]), with no differences between the groups (OR: 1.15; 95%CI: 0.69–1.80).

### Subgroup analyses

**Figure 4:**
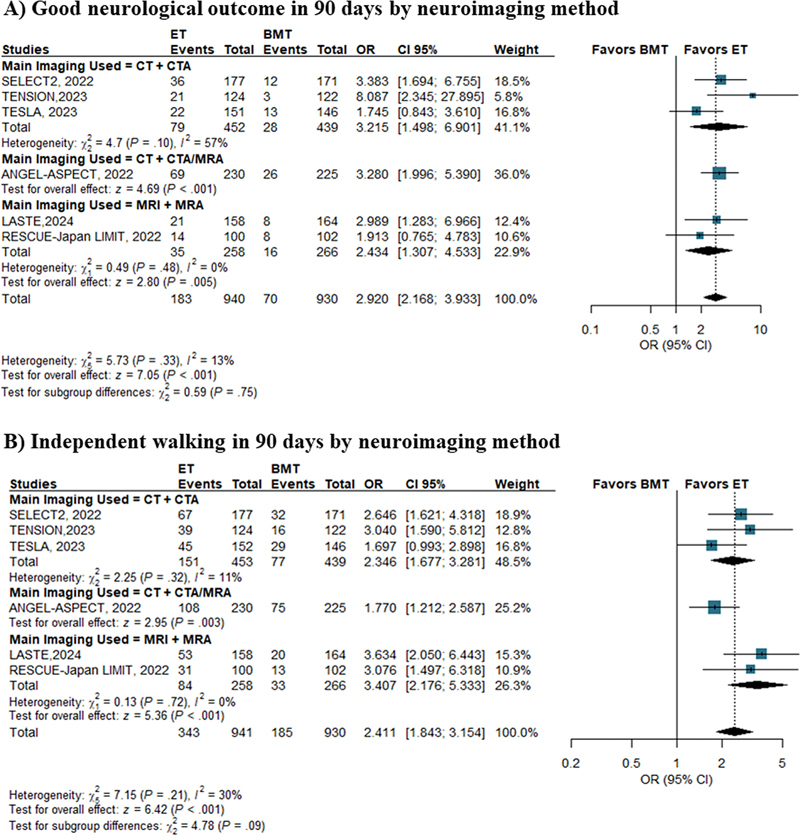
Abbreviations: 95% CI, 95% confidence interval; BMT, best medical treatment; CT, non-contrast computed tomography; CTA, computed tomography angiography; ET, endovascular thrombectomy; OR, odds ratio; MRA, magnetic resonance angiography; MRI, magnetic resonance imaging. ( **A** ) Good neurological outcome after 90 days according to the neuroimaging method. ( **B** ) Independent walking after 90 days according to the neuroimaging method.

Even among the subgroup of patients selected for ET primarily by brain computed tomography (CT), ET yielded a higher frequency of good neurological outcomes (OR: 3.21; 95%CI: 1.49–6.90; Figure 4A ) and independent walking (OR: 2.34; 95%CI: 1.68–3.28; Figure 4B ). Another TSA which included only trials that used plain brain CT with CT angiography (CTA) as the main imaging modality was conducted, and it confirmed statistical significance considering a power of 90%. However, the sample size was insufficient for a very conservative power of 99% ( **Supplementary Material 2** , **Figures S4** – **S5** ; online only).

**Figure 5:**
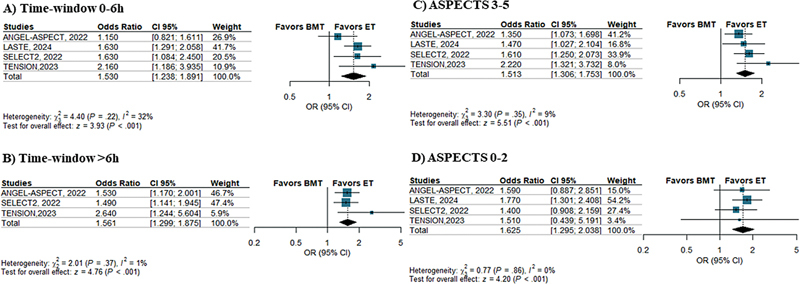
Abbreviations: 95% CI, 95% confidence interval; BMT, best medical treatment; ET, endovascular thrombectomy; OR, odds ratio. Subgroup analysis according to the time-window since symtpms onset and baseline ASPECTS.

Subgroups of patients were dichotomized according to time-to-randomization (≤ 6 hours and > 6 hours from symptom onset) for the ordinal shift in the mRS scores. Endovascular thrombectomy was associated with higher odds of good neurological outcome in both subgroups; ≤ 6 hours from symptom onset (OR: 1.53; 95%CI: 1.24–1.89; Figure 5A ) and > 6 hours from symptom onset (OR: 1.56; 95%CI: 1.3–1.87; Figure 5B ). Endovascular thrombectomy was also associated with good neurological outcomes among subgroups of patients with ASPECTS ranging from 3 to 5 (OR: 1.51; 95%CI: 1.30–1.75; Figure 5C ), as well as those with ASPECTS ranging from 0 to 2 (OR: 1.62; 95%CI: 1.29–2.03; Figure 5D ).

### Quality assessment

The risk of bias assessment indicated a low risk of bias for all studies. While subjects and providers were not blinded to treatment allocation, the included studies 9 10 11 12 13 14 used appropriate randomization methods, with little missing data, and the assessment of data was undertaken by blinded investigators ( **Supplementary Material 2, Table S1** ; online only). A funnel plot analysis showed symmetrical distribution according to weight, indicating a low risk of publication bias ( **Supplementary Material 2, Figures S6,S7** ; online only).

## DISCUSSION

In the current systematic review and meta-analysis of 6 RCTs 9 10 11 12 13 14 including 1,887 patients, ET was compared with BMT in patients with large-core stroke presenting within the first 24 hours of symptom onset. To the best of our knowledge, the present is the first meta-analysis using official, peer-reviewed, published data from most trials on this topic. Our main findings were that ET was associated with higher odds of good neurological outcomes (mRS score ≤ 2), independent walking (mRS score ≤ 3), ENI, a shift toward better outcomes in the distribution of mRS scores at 90 days, and a higher chance of intracranial bleeding and symptomatic intracranial bleeding; moreover, mortality at 90 days and the rate of decompressive craniectomy were not different between groups. The subgroup analysis indicated that non-contrast head CT with CTA is an appropriate method for screening and selecting large core stroke patients for ET.

The benefits of ET appear to persist in the long term, as demonstrated by the recently-published 26 1-year follow-up of the SELECT2 for shift in mRS scores (OR: 1.43; 95%CI: 1.14–1.78). Moreover, the authors 26 reported no difference in mortality at the 1-year follow-up.

Endovascular thrombectomy was associated with ENI, with moderate heterogeneity. The heterogeneity could be explained by the different time points at which ENI was assessed (between 24 hours and 6 days after randomization). We concluded that comparison of ENI outcomes between 24 hours to 6 days was acceptable, because it is a considerably shorter follow-up time compared with the analyses of good neurological outcomes and independent walking, which were conducted 90 days after randomization.

Subgroup analyses based on the main modality of neuroimaging for patient selection consistently favored ET, although moderate heterogeneity was observed. The TESLA 12 and TENSION 13 trials used mostly non-contrast head CT with CTA for patient selection; the SELECT2 10 used a mixed criteria of ASPECTS < 6 and ischemic core > 50 mL on perfusion imaging; the ANGEL-ASPECT 11 used a combination of CT, CTA, and magnetic resonance angiography (MRA); the RESCUE-Japan LIMIT 9 and LASTE 14 used MRI and MRA. Non-contrast head CT with CTA appeared to be a satisfactory approach to screen and select appropriate patients to receive ET; however, the heterogeneity among studies was high (I ^2^ = 57%). This was probably due to the smaller sample size of the subgroup analysis ( *n* = 891) compared with that of the total analysis ( *n* = 1,870). To confirm statistical significance, we performed a separate TSA for the modality of neuroimaging used to select the patients for ET. The TSA indicated that the sample size was insufficient for a very conservative power of 99% ( **Supplementary Material 2** , **Figures S4** – **S5** ; online only). Thus, while our impression is that plain brain CT with CTA are appropriate to select patients for ET in this population, larger sample sizes are needed to deliver a conclusive finding. We noted that there is one relevant ongoing trial, 27 and we anticipate the potential for further analyses that may confirm our results.

There is an ongoing debate as to whether ET is cost-effective for the treatment of patients with large-core stroke. A previous cost-effectiveness study 28 conducted within the Brazilian public health system favored ET over BMT for LVO and low- to mid-core stroke. Another study 29 reported results favoring ET in the treatment of large-core stroke based on cost-effectiveness data from the RESCUE-Japan LIMIT 9 and published health-economic data from Europe. Alongside the current findings indicating similar outcomes regarding non-contrast head CT with CTA and other less accessible/more expensive neuroimaging modalities, these cost-effectiveness studies further support the feasibility of ET for the treatment of large-core stroke, even in lower-income centers. However, a more comprehensive economic cost-benefit analysis is crucial to contextualize its efficacy. Understanding the financial implications of the intervention will enhance decision-making processes and ensure optimal allocation of resources for improved healthcare outcomes.

Subgroup analyses conducted among groups with low (3–5) and very low (0–2) ASPECTS showed that ET had higher odds of a better mRS score compared with BMT in these subpopulations. Notably, the benefit of ET among patients with very low ASPECTS is of particular clinical relevance, because these patients can be systematically selected for ET treatment based on their higher odds of better functional outcomes.

Subgroup analyses stratified by > 6 hours and ≤ 6 hours from symptom onset (or last time seen well) showed that, in both groups, ET was superior to BMT. One of the trials 13 included patients treated within 12 hours of symptom onset, while two other trials 11 12 included patients treated any time within the first 24 hours of symptom onset. Of note, the weight of the individual study 13 with shorter time-to-randomization was low (5.9%) and did not significantly change the proportion of variability (I ^2^ = 1%).

Mortality did not differ between the ET and BMT groups. This was consistent with the results of the individual studies, with the exception of LASTE, 14 which reported a significant reduction in mortality in the ET group (OR: 0.45; 95%CI: 0.29–0.70), and TENSION, 13 which reported a significant difference in mortality favoring the ET group. This may be explained because the TENSION trial used an adjusted logistic regression model that considered the randomization stratification factors (symptom onset < 6 hours or > 6 hours, and National Institutes of Health Stroke Scale [NIHSS] score < 18 or 19–25), while the current analysis only considered the total number of events, without adjustments. Mortality in the LASTE trial may be explained by the lower time window used (6.5 hours).

Predictably, the literature reports 30 a lower proportion of hemicraniectomy (2.8%) among non-large-core strokes studies compared with that of the large-core stroke studies included in the current review. 9 10 11 12 13 14 However, decompressive craniectomy was uncommon throughout the included studies, with 72/769 (9.36%) patients undergoing decompressive craniectomy in the BMT group compared with 85/765 (11.1%) in the ET group. While we found no significant difference between the groups, considering the small sample size, the results of the present study should be interpreted with caution. Lower baseline ASPECTS is a risk factor for increased intracranial pressure, symptomatic malignant cerebral edema, and severe intracranial hemorrhage. 31 32 Considering that all patients in the current review presented low ASPECTS when compared with the pivotal thrombectomy studies, larger samples and individual data analyses on specific risk factors in this population are needed.

Intracranial bleeding and symptomatic intracranial bleeding were more common in the ET group than the BMT group. Intracranial bleeding presented a high heterogeneity, and subsequent sensitivity analyses showed that the ANGEL-ASPECT 11 and RESCUE-Japan LIMIT 9 trials were independently responsible for the statistical significance in both instances. The literature presents conflicting reports about the risk of intracranial bleeding among the Asian population. Consequently, in Japan, a lower dose of alteplase is used for the treatment of ischemic stroke. The findings of a clinical trial reporting non-inferiority of neurological outcomes and lower bleeding risks 33 were the basis for the Japanese guidelines. 34 Moreover, observational studies and a systematic review 35 36 37 have reported a higher risk of hemorrhagic stroke among Asian patients. On the other hand, a comparative study 38 between North American (60.5% Caucasian, 19.8% African-American, 5.8% Asian, and 13.9% of other races) and Chinese (100% East Asian) patients with ischemic stroke reported no differences in hemorrhagic transformations, although the population comorbidities and ages were not well balanced between the groups. Despite the increased risk of bleeding among patients in the ANGEL-ASPECTS and RESCUE-Japan-LIMIT, the results indicate that ET remains a viable option for the treatment of patients with large-core stroke.

The current meta-analysis has some limitations: the methodological differences among the included studies limits the generalizability of our findings; other subgroup analyses could not be performed (such as those regarding thrombolysis, coagulopathy, stroke etiology etc.) because of differences in the manner in which these outcomes were reported, or even due to the absence of data; the small number of studies included in the analysis limited the use of robust statistical tools such as meta-regression; and we were unable to access individual-level data which could have yielded more insights into the profiles of patients who may benefit the most from ET.

Of note, the TESLA 12 study awaits peer-review for publication. After its publication, if the results differ from those of the current version, the present meta-analysis will be reassessed, and the updated interpretation of our results will be promptly submitted for amendment. Given the missing evidence, future studies could address: if the outcomes might shift according to the age of the patients; a combined analysis between ASPECTS and the time since symptom onset (or last time seen well); the limitations of ASPECTS interpretations; and core volume evaluation (with advanced neuroimaging). Data on individual patients could help resolve some of this missing information.

In patients with large-core ischemic stroke, ET performed within the first 24 hours of symptom onset is associated with a better chance of good neurological outcome, independent walking, and ENI, compared with BMT. However, there is a higher risk of intracranial bleeding and symptomatic intracranial bleeding associated with ET. Non-contrast head CT with CTA seems to be an appropriate method to screen and select patients to receive ET, but more studies are needed to reach the sample size required for a definitive conclusion.
